# Enhancing intracranial efficacy prediction of osimertinib in non-small cell lung cancer: a novel approach through brain MRI radiomics

**DOI:** 10.3389/fneur.2024.1399983

**Published:** 2024-08-30

**Authors:** Xin Tang, Yuan Li, Wen-Lei Qian, Pei-Lun Han, Wei-Feng Yan, Zhi-Gang Yang

**Affiliations:** Department of Radiology, West China Hospital, Sichuan University, Chengdu, China

**Keywords:** MRI radiomics, osimertinib, intracranial efficacy, non-small cell lung cancer, predictive model

## Abstract

**Introduction:**

Osimertinib, a third-generation EGFR-TKI, is known for its high efficacy against brain metastases (BM) in non-small cell lung cancer (NSCLC) due to its ability to penetrate the blood–brain barrier. This study aims to evaluate the use of brain MRI radiomics in predicting the intracranial efficacy to osimertinib in NSCLC patients with BM.

**Materials and methods:**

This study analyzed 115 brain metastases from NSCLC patients with the EGFR-T790M mutation treated with second-line osimertinib. The primary endpoint was intracranial response, and the secondary endpoint was intracranial progression-free survival (iPFS). We performed tumor delineation, image preprocessing, and radiomics feature extraction. Using a 5-fold cross-validation strategy, we built radiomic models with eight feature selectors and eight machine learning classifiers. The models’ performance was evaluated by the area under the receiver operating characteristic curve (AUC), calibration curves, and decision curve analysis.

**Results:**

The dataset of 115 brain metastases was divided into training and validation sets in a 7:3 ratio. The radiomic model utilizing the mRMR feature selector and stepwise logistic regression classifier showed the highest predictive accuracy, with AUCs of 0.879 for the training cohort and 0.786 for the validation cohort. This model outperformed a clinical-MRI morphological model, which included age, ring enhancement, and peritumoral edema (AUC: 0.794 for the training cohort and 0.697 for the validation cohort). The radiomic model also showed strong performance in calibration and decision curve analyses. Using a radiomic-score threshold of 199, patients were classified into two groups with significantly different median iPFS (3.0 months vs. 15.4 months, *p* < 0.001).

**Conclusion:**

This study demonstrates that MRI radiomics can effectively predict the intracranial efficacy of osimertinib in NSCLC patients with brain metastases. This approach holds promise for assisting clinicians in personalizing treatment strategies.

## Introduction

Lung cancer remains the leading cause of cancer-related deaths globally (1). Among the various types, non-small cell lung cancer (NSCLC) is the most prevalent, accounting for approximately 85% of all lung cancer cases (2). The incidence of brain metastasis in NSCLC is 20–30% (3, 4). Notably, the risk of brain metastases in NSCLC patients with mutant epidermal growth factor receptor (EGFR) is significantly higher than that in those with wild-type EGFR (5, 6). Brain metastasis can cause severe neurological and cognitive dysfunction, which remarkably threatens patients’ quality of life and shortens survival time (7).

First-and second-generation EGFR-tyrosine kinase inhibitors (EGFR-TKIs, e.g., gefitinib, erlotinib, and afatinib) are standard treatments for NSCLC patients with sensitive EGFR mutations. Nevertheless, due to the high affinity of these agents to efflux transporters, they have a low blood–brain barrier penetrating rate (8–10). As a result, brain metastasis is one of the most common metastatic sites of NSCLC patients after progression on first-line EGFR-TKI therapy. EGFR-T790M mutation is the main cause of treatment failure of the first-line EGFR-TKI. Approximately 50–60% of NSCLC patients developed EGFR-T790M mutation after resistance to the first-line EGFR-TKI (11–13). Osimertinib, as a third-generation EGFR-TKI, can not only inhibit the EGFR-T790M mutation but also exert high antitumor activity for the central nervous system (CNS) (10). The previous studies demonstrated that for patients with brain metastasis, osimertinib showed an intracranial efficacy superior to that of chemotherapy (14, 15) and first−/s-generation EGFR-TKIs (16), with an intracranial objective response rate (ORR) being 54–71% (14, 15, 17, 18). In 2018, the FLAURA trial demonstrated that osimertinib could be effectively used as a first-line therapy, and it is gradually becoming the standard of care for initial treatment (19). However, it cannot be neglected that a substantial proportion of brain metastases still could not benefit from osimertinib therapy. Accurate screening of candidates sensitive to osimertinib treatment is the key to guiding individualized treatment for brain metastatic NSCLC patients.

Radiomics is an emerging research field that converts medical images into quantitative data by extracting radiomic features, such as pixel intensity, shape, texture, etc., from tumor regions (20). Compared with the qualitative morphological features of traditional images, high-throughput radiomic features can more comprehensively and objectively reflect tumor heterogeneity invisible to the naked eye. In addition, since radiomics is the additional processing of acquired medical images, it does not increase the financial and physical burden on patients. Recently, with the support of continuously optimized machine learning algorithms, radiomics-related research has developed rapidly. It has been reported that brain MRI radiomics has a strong ability to predict the intracranial efficacy of treatments, including radiotherapy, ALK-TKI targeted therapy, and immunotherapy in lung cancer patients with brain metastases (21–26). Additionally, some studies have attempted to analyze the predictive value of MRI radiomics for the efficacy of first-and second-generation EGFR-TKIs in treating brain metastases (27). Another study reported on the effectiveness of MRI radiomics in predicting treatment outcomes for brain metastases using first-and second-line Osimertinib, either alone or in combination with chemotherapy and anti-angiogenic therapy (28). However, the conclusions of this study were limited by the heterogeneous patient population.

Therefore, the purpose of this study was to explore the predictive effect of brain MRI radiomics on the intracranial efficacy of osimertinib treatment in advanced NSCLC patients with brain metastases after the failure of the first-line EGFR-TKI.

## Methods

### Patients

**Figure 1:**
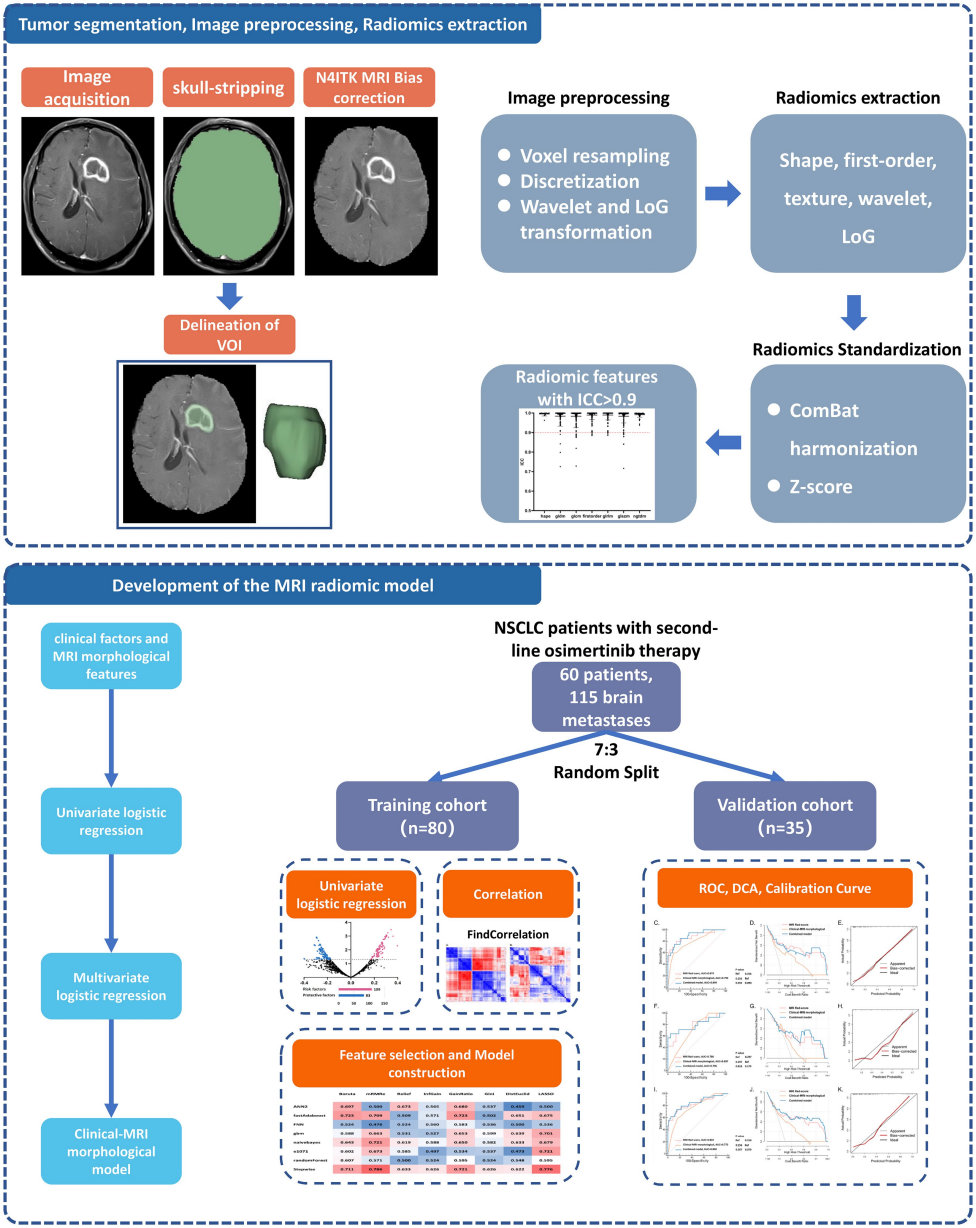
Analysis workflow of this study. The upper panel shows the process of tumor segmentation, image preprocessing, and radiomics extraction. The lower panel exhibits the details of model development and validation. NSCLC, non-small cell lung cancer; VOI, volume of interest; ICC, interclass correlation coefficients; ROC, receiver operator characteristic curve; DCA, decision curve analysis.

This study included 60 EGFR-T790M-positive NSCLC patients with brain metastases (115 brain metastases in total) who received second-line osimertinib treatment from West China Hospital. The overall study design is detailed in Figure 1. This retrospective study was approved by the Biomedical Research Ethics Committee of West China Hospital, Sichuan University, and patients’ written informed consent was waived.

The main inclusion criteria included: (1) patients with advanced NSCLC diagnosed by histopathology and imaging examinations, classified as stage IV according to the American Joint Committee on Cancer (AJCC) 8th edition TNM staging system; (2) patients previously treated with first-line EGFR-TKI therapy (first/s-generation EGFR-TKI) who had progressive disease (RECIST 1.1 criteria); (3) patients with EGFR-T790M mutation after first-line EGFR-TKI therapy and received sequential second-line osimertinib treatment; (4) patients had contrast-enhanced brain MRI images within 1 month before osimertinib treatment; (5) patients had ≥1 measurable brain metastases before osimertinib treatment (RECIST 1.1 criteria). Main exclusion criteria of cohort 1: (1) patients with missing or poor-quality brain MRI images; (2) patients with leptomeningeal metastases or brain metastases with unclear margins; (3) patients with incomplete clinical data; (4) patients who received other anti-tumor treatments such as radiotherapy and chemotherapy during osimertinib treatment. Data collection was from September 2015 to November 2021. The last follow-up was in December 2021.

### Scanning parameters of brain MRI

Patients included in the study were scanned with one of the following 5 MRI machines: Siemens Avanto (1.5 T), UIH UMR588 (1.5 T), TOSHIBA MRT200SP5 (1.5 T), GE discovery MR750W (3.0 T) and Philips achieva (3.0 T). All patients had brain MRI contrast-enhanced T1-weighted (T1CE) images. In line with previous studies, MRI radiomics features were extracted based on T1CE images in the current study (24, 29, 30). The acquisition parameters of T1CE images are as follows: Siemens: repetition time/echo time (TR/TE) = 2040/3.94 ms, flip angle = 15°, matrix = 256 × 224, slice thickness = 1 mm, pixel size = 1 × 1 mm^2^; UIH: TR/TE = 13.83/5.9 ms, flip angle = 10°, matrix = 256 × 232, slice thickness = 1 mm, pixel size = 1 × 1 mm^2^; GE: TR/TE = 2383/20.78 ms, flip angle = 142°, matrix = 320 × 256, slice thickness = 5 mm, pixel size = 0.4688 × 0.4688 mm^2^; TOSHIBA: TR/TE = 580/8.0 ms, flip angle = 90°, matrix = 320 × 160, slice thickness = 6 mm, pixel size = 0.6875 × 0.6875 to 0.75 × 0.75 mm^2^; Philips: TR/TE = 130/2.3 ms, flip angle = 80°, matrix = 256 × 205, slice thickness = 6 mm, pixel size = 0.4492 × 0.4492 mm^2^. After rapid intravenous injection of gadolinium-DTPA contrast agent (dose 0.1 mmol/kg, flow rate 2–3 mL/s), enhanced brain MRI scans were performed, and T1CE images were obtained.

### MRI morphological manifestations

Two radiologists evaluated the MRI morphological manifestations of brain metastases, and disagreements were solved through discussion. Both radiologists were blinded to patients’ treatment responses to osimertinib. MRI morphological manifestations include: (1) the number and largest diameter of brain metastases; (2) the location of brain metastases (frontal lobe, parietal lobe, occipital lobe, temporal lobe, cerebellum, and others); (3) the presence or absence of edema around brain metastases (peritumor edema); (4) enhancement pattern (with or without ring enhancement).

### Clinical factors of patients

This study retrospectively collected clinical data of all patients, including age, gender, smoking history, Eastern Cooperative Oncology Group performance status (ECOG-PS) score, clinical TNM stage, initial EGFR mutation, first-line EGFR-TKI agent, and patients’ progression-free survival (PFS) in first-line EGFR-TKI therapy. The clinical stage was assessed according to the AJCC 8th edition TNM staging system. PFS of first-line EGFR-TKI therapy was defined as the interval from initiation of treatment to radiographic progression (RECIST 1.1 criteria).

### Study endpoints

Intracranial efficacy of osimertinib on each individual brain metastatic lesion was assessed according to RECIST 1.1 criteria. Intracranial objective response (favorable intracranial treatment responses) included complete response (CR) and partial response (PR), while poor intracranial treatment responses were stable disease (SD) and progressive disease (PD). To determine if radiomics features can predict unfavorable therapeutic responses in patients undergoing Osimertinib treatment, we selected SD/PD as the endpoints, and the secondary endpoint was intracranial progression-free survival (iPFS) which refers to the interval from the start of osimertinib to intracranial tumor progression.

### Delineation of the volume of interest

The brain MRI images obtained by the Picture Archiving and Communication System (PACS) were uploaded to the open-source software 3D slicer in DICOM format (Version 4.10.2).^1^ Two radiologists independently and manually outlined the VOI of brain metastases on the axial T1CE images. Up to five brain metastases were selected as target lesions in patients with multiple brain metastases (31). If the number exceeds five, the largest five brain metastases were selected for delineation (32).

### Image preprocessing and radiomic feature extraction

The image preprocessing and radiomics feature extraction methods applied in this study are in line with the recommendations of the Image Biomarkers Standardization Initiative (IBSI).^2^ To minimize differences in image acquisition parameters between different MRI scanning machines and improve reproducibility, we performed image preprocessing before radiomic feature extraction, mainly including five steps: (1) Use the “Swiss Skull Stripper” module in the 3D slicer software to perform the skull stripping of the T1CE image. (2) Use the “N4ITK MRI Bias correction” module to perform bias field correction on the image to eliminate the inhomogeneity of low-frequency intensity. (3) To standardize the voxel spacing, the sitkBSpline interpolation algorithm is used to resample all MRI voxels (1 × 1 × 1 mm^3^). (4) The image signal intensity is normalized by grayscale discretization (bin width = 25HU) uniform processing. (5) Apply Wavelet and Laplacian of Gaussian (LoG) filter transformation (sigma: 1.0–2.5) to remove interference signals.

After image preprocessing, the Python package “PyRadiomics” (33)^3^ embedded in the 3D-slicer software was used to extract radiomic features from the VOI of each delineated brain metastatic lesion. Totally, 1,223 radiomic features were automatically extracted, including 107 original features, 744 Wavelet features, and 372 LoG filtered transformation features. These radiomic features were grouped into three categories: 14 shape features, 234 first-order features, and 975 texture features. Texture features included gray-level cooccurrence matrix (GLCM, *N* = 312), gray-level run length matrix (GLRLM, *N* = 208), gray-level difference matrix (GLDM, *N* = 182), gray-level size zone matrix (GLSZM, *N* = 208), and neighborhood gray tone difference matrix (NGTDM, *N* = 65).

After extracting the radiomic features, to minimize the multi-machines effect, we performed the ComBat harmonization (34),^4^ a widely used algorithm to correct the batch effect of radiomics extracted by different scanners (35). We also applied Z-score transformation to normalize the radiomic features.

### Preliminary selection of predictive radiomic features

To evaluate the consistency of the radiomic features extracted by the two researchers, we calculated the inter-observer interclass correlation coefficients (ICC) for each radiomic feature. An ICC value>0.9 was considered as the criterion of high reproducibility. The preliminary selection of predictive radiomics features consisted of two steps: (1) univariate logistic analysis was used to initially screen out potential predictors; (2) considering the high degree of collinearity within radiomic features, which may lead to overfitting of the radiomic model, we performed a correlation analysis on the radiomic features and excluded redundant features through the findCorrelation function in the “caret” R package.

### Construction of radiomic model

A total of 115 measurable brain metastases were randomly split into the training (*n* = 80) and validation (*n* = 35) groups (ratio: 7:3). To reduce the bias from cluster correlation, we assigned the multiple brain metastases from the same patient simultaneously to either the training or the validation group as the previous study did (24, 36).

Eight feature selectors and eight classifier machine learning algorithms were used to construct radiomic models for predicting the intracranial efficacy of second-line osimertinib therapy. A nested 5-fold cross-validation strategy was applied. Eventually, a total of 64 (8 × 8) combinations of radiomic feature selectors and classifiers were generated.

In selecting the feature selectors, Boruta was chosen for its comprehensive approach to identifying all relevant features, ensuring that no potential predictive feature is overlooked. Minimum Redundancy Maximum Relevance (mRMR) was included due to its balanced approach in selecting features that provide the most information while minimizing redundancy, which is crucial for enhancing model performance. LASSO was selected for its dual functionality in variable selection and regularization, which helps in managing overfitting and improving the model’s generalizability. Relief was chosen for its ability to capture feature dependencies and interactions, which is often beneficial in complex datasets. InfGain and GainRatio were utilized for their effectiveness in measuring the importance of features based on information gain, which is essential for building robust models. Gini was included for its simplicity and proven effectiveness in feature selection tasks. DistEuclid was chosen for its straightforward approach in selecting features based on Euclidean distance, aiding in identifying the most relevant features for classification tasks.

Regarding the classifiers, the artificial neural network (ANN) was chosen for its powerful ability to model complex nonlinear relationships in data, making it suitable for the intricate patterns found in radiomic data. Adaptive boosting (Adaboost) was selected for its capability to improve the performance of weak classifiers, thereby enhancing the overall predictive accuracy of the model. Fast nearest neighbor (FNN) was included for its efficiency and effectiveness in classification tasks, particularly when dealing with high-dimensional data. Gradient boosting decision tree (GBDT) was chosen for its strong performance in handling structured data and its ability to produce high-accuracy models through iterative boosting. Naive bayes was selected for its simplicity and effectiveness in probabilistic classification, making it a robust choice for many classification tasks. Support vector machine (SVM) was included for its effectiveness in high-dimensional spaces and its ability to construct hyperplanes for classification tasks. Random forest (RF) was chosen for its robustness in handling large datasets with high dimensionality and its ability to prevent overfitting through ensemble learning. Stepwise logistic regression (SLR) was included for its simplicity, interpretability, and effectiveness in linear classification tasks.

The main R packages involved were as follows: “Boruta,” “mRMRe,” “e1071,” “naivebayes,” “CORElearn,” “ANN2,” “fastAdaboost,” “FNN,” “gbm,” and “glmnet.” The predictive accuracy of the machine learning model was assessed by the area under the receiver operator characteristic curve (AUC of the ROC). The combination of feature selector and classifier with the highest AUC was used to construct the optimal radiomics model. An MRI radiomic score (Rad-score) was constructed based on the final model.

### Evaluation of radiomic model

This study evaluated the performance of the predictive model from three different aspects: (1) AUC of ROC was used to reflect the predictive accuracy or discrimination power of the model. Delong test was used to compare the AUC of distinct ROCs; (2) Calibration curves were used to assess the consistency (goodness of fit) between the predictive and actual probability of poor intracranial response (SD/PD). (3) Decision curve analysis (DCA) was used to visualize the clinical utility of predictive models by quantifying the net benefit at different threshold probabilities. iPFS is an important long-term indicator reflecting the efficacy of intracranial treatment. To further explore whether a radiomic model can predict iPFS in patients treated with osimertinib, we used the X-tile software to determine the optimal cut-off point for the model’s prediction of iPFS.

### Statistical software

SPSS 25.0 software and R software (version: 4.0.5) were used for statistical analysis. All statistical tests were two-sided. In the stepwise logistic regression analysis, a *p* < 0.1 was used as the standard for variable exclusion. A *p* < 0.05 was considered statistically significant in all the other tests.

## Results

### Patients and brain metastases

Among the 60 EGFR-T790M-positive NSCLC patients who received second-line osimertinib therapy, 40 (66.7%) had multiple brain metastases. Totally, 115 brain metastases were included in the analysis. We randomly split the 115 brain metastases into the training (*N* = 80) and the validation (*N* = 35) cohort with a ratio of 7:3 (Table 1; Supplementary Table S1). To reduce bias due to cluster correlation, brain metastases from the same patient were assigned to the same group, as recommended by previous studies (24, 36). The baseline characteristics of the two groups were well balanced.

**Table 1:** Baseline characteristics of NSCLC patients receiving second-line osimertinib therapy in the training and validation cohort.

	Total cohort		Training cohort		Validation cohort		*P-*value
	N	%	N	%	N	%	
Total number of BM	115	100.0%	80	69.6%	35	30.4%	–
Number of BM							
1	20	33.3%	13	35.1%	7	30.4%	0.707
≥2	40	66.7%	24	64.9%	16	69.6%	
Age							
≤60 Y	27	45.0%	16	43.2%	11	47.8%	0.936
>60 Y	33	55.0%	21	56.8%	12	52.2%	
Sex							
Female	35	58.3%	22	59.5%	13	56.5%	1.000
Male	25	41.7%	15	40.5%	10	43.5%	
Smoking history							
No	54	90.0%	33	89.2%	21	91.3%	1.000
Yes	6	10.0%	4	10.8%	2	8.7%	
ECOG-PS score							
0	30	50.0%	19	51.4%	11	47.8%	1.000
≥1	30	50.0%	18	48.6%	12	52.2%	
Initial EGFR mutation							
L858R	24	40.0%	14	37.8%	10	43.5%	0.900
19Del	30	50.0%	19	51.4%	11	47.8%	
Others	6	10.0%	4	10.8%	2	8.7%	
First-line EGFR-TKI drug							
Gefitinib	33	55.0%	20	54.1%	13	56.5%	0.979
Erlotinib	11	18.3%	7	18.9%	4	17.4%	
Icotinib	14	23.3%	9	24.3%	5	21.7%	
Afatinib	2	3.3%	1	2.7%	1	4.3%	
First-line EGFR-TKI PFS							
<12 Mo	23	38.3%	14	37.8%	9	39.1%	1.000
≥12 Mo	37	61.7%	23	62.2%	14	60.9%	
T stage							
T1	10	16.7%	6	16.2%	4	17.4%	0.887
T2	13	21.7%	8	21.6%	5	21.7%	
T3	5	8.3%	3	8.1%	2	8.7%	
T4	32	53.3%	20	54.1%	12	52.2%	
N stage							
N0	12	20.0%	9	24.3%	3	13.0%	0.472
N1	7	11.7%	4	10.8%	3	13.0%	
N2	24	40.0%	14	37.8%	10	43.5%	
N3	17	28.3%	10	27.0%	7	30.4%	
M stage							
M1b	8	13.3%	4	10.8%	4	17.4%	0.735
M1c	52	86.7%	33	89.2%	19	82.6%	
Stage							
IVa	8	13.3%	4	10.8%	4	17.4%	0.735
IVb	52	86.7%	33	89.2%	19	82.6%	

NSCLC, Non-Small Cell Lung Cancer; BM, brain metastases; PFS, Progression-free survival; EGFR, Epidermal Growth Factor Receptor; TKI, Tyrosine Kinase Inhibitor; ECOG-PS, Eastern Cooperative Oncology Group performance score.

The overall intracranial efficacy of the second-line osimertinib is shown in Supplementary Figure S1. In aggregated, 9 (7.8%), 72 (62.6%), 30 (26.1%), and 4 (3.5%) brain metastases achieved the best therapeutic response of CR, PR, SD, and PD, respectively. The objective response rate (CR + PR) of brain metastases was 81/115 (70.4%). At the end of follow-up, the median iPFS was 15.4 months (95% CI: 14.4–23.9 months) for the total cases.

### Predictive value of clinical factors and MRI morphological features

Univariate logistic regression was performed to explore the predictive value of clinical factors and MRI morphological features on the intracranial efficacy of the second-line osimertinib (Table 2A). Among clinical factors, only age > 60 years (OR: 4.06, 95%CI: 1.64–10.05, *p* = 0.002) was associated with an unfavorable intracranial response (SD/PD). In MRI morphological features, ring enhancement (OR: 2.53, 95%CI: 1.12–5.75, *p* = 0.026) and peritumoral edema (OR: 4.72, 95%CI: 1.95–11.45, *p* = 0.001) predicted high risk of SD/PD. Other parameters were not related to the intracranial efficacy of osimertinib. Multivariate analysis further revealed that age, ring enhancement, and peritumoral edema were independent prognosticators (Table 2B). The AUC of the predictive model established based on these three factors was 0.773 for the total patients.

**Table 2:** The value of various clinical factors and MRI morphologic features in predicting the intracranial therapeutic efficacy of the second-line osimertinib treatment.

A. Univariate logistic regression	OR	95% CI OR	*p-*value
Age: >60 vs. ≤60 Y	4.06	1.64–10.05	0.002
Sex: Male vs. Female	0.77	0.34–1.75	0.539
Smoking history: Yes vs. No	0.85	0.25–2.88	0.792
ECOG-PS score: ≥1 vs. 0	0.86	0.40–1.83	0.692
Initial EGFR mutation: 19Del vs. L858R	0.74	0.32–1.70	0.473
Initial EGFR mutation: Others vs. L858R	0.43	0.08–2.21	0.314
First-line EGFR-TKI: Erlotinib vs. Gefitinib	0.48	0.14–1.64	0.245
First-line EGFR-TKI: Icotinib vs. Gefitinib	0.53	0.20–1.43	0.209
First-line EGFR-TKI: Afatinib vs. Gefitinib	0.91	0.08–10.6	0.939
First-line EGFR-TKI PFS: ≥12 Mo vs. <12 Mo	1.83	0.81–4.14	0.148
T stage: T3-4 vs. T1-2	0.90	0.39–2.06	0.805
N stage: N0-1 vs. N2-3	1.17	0.48–2.87	0.733
M stage: M1c vs. M1b	0.38	0.10–1.42	0.150
Stage: IVb vs. IVa	0.38	0.10–1.42	0.150
Maximum diameter of BM: >2 cm vs. ≤2 cm	1.57	0.48–5.21	0.465
Ring enhancement: Yes vs. No	2.53	1.12–5.75	0.026
Peritumor edema: Yes vs. No	4.72	1.95–11.45	0.001
Location of BM: Frontal lobe vs. Parietal lobe	4.67	0.89–24.35	0.068
Location of BM: Occipital lobe vs. Parietal lobe	2.40	0.41–14.11	0.333
Location of BM: Temporal lobe vs. Parietal lobe	4.67	0.78–28.05	0.092
Location of BM: Cerebellum vs. Parietal lobe	0.40	0.03–4.96	0.476
Location of BM: Others vs. Parietal lobe	2.00	0.31–13.06	0.469

B. Multivariate logistic regression	OR	95% CI OR	*p-*value
Age: >60 vs. ≤60 Y	4.23	1.61–11.25	0.003
Ring enhancement: Yes vs. No	2.337	0.92–5.94	0.074
Peritumor edema: Yes vs. No	3.80	1.48–9.77	0.006

* Stepwise regression method, exclusion criteria for variables: *p* < 0.1.

NSCLC, Non-Small Cell Lung Cancer; ECOG-PS, Eastern Cooperative Oncology Group performance score; EGFR, Epidermal Growth Factor Receptor; TKI, Tyrosine Kinase Inhibitor; PFS, Progression-free survival; BM, brain metastases.

### Development of the MRI radiomic model

**Figure 2:**
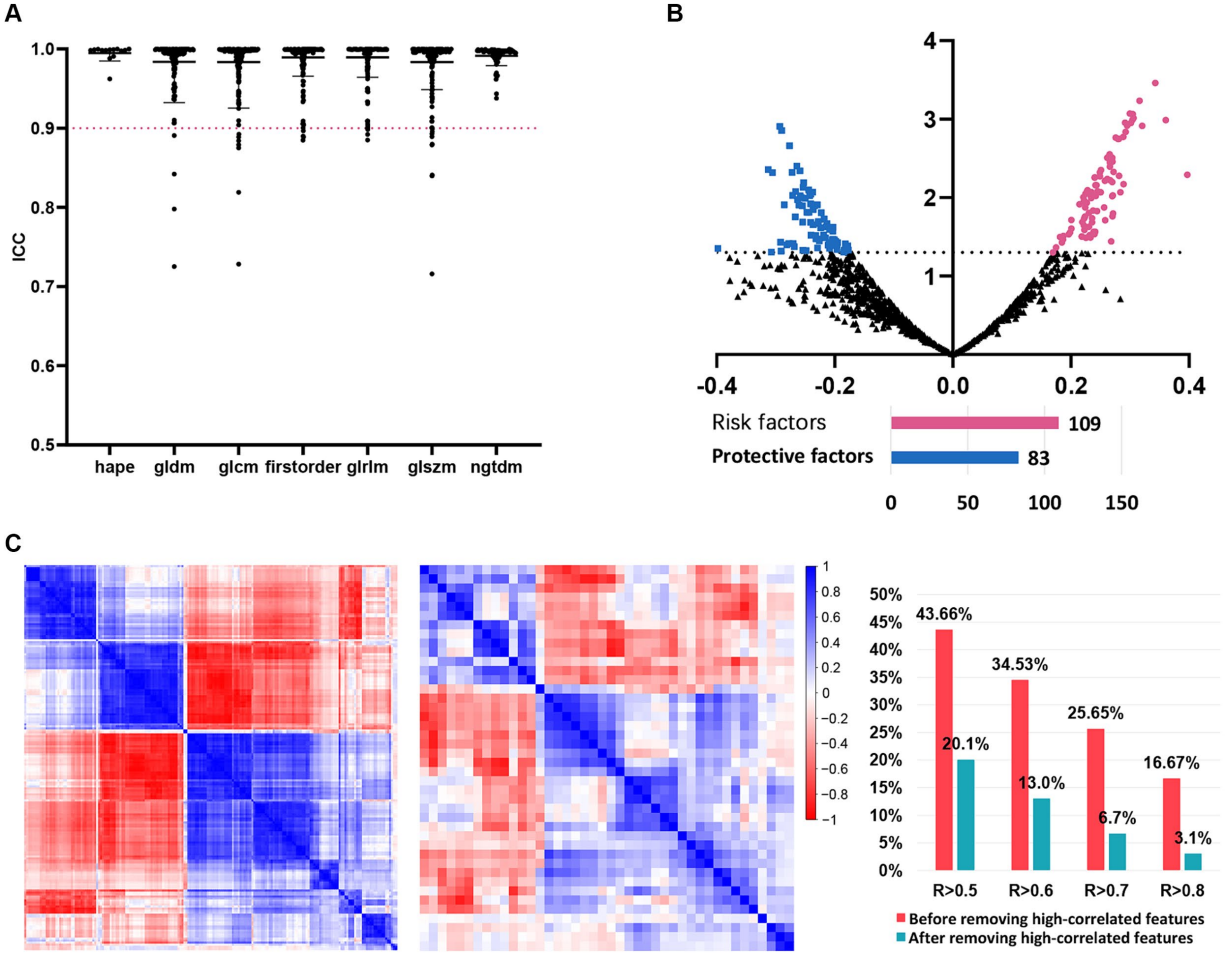
The detailed process of radiomic features before models’ construction. **(A)** ICC analysis. Radiomic features with ICC > 0.9 were included in further analysis. **(B)** Univariate logistic analysis evaluating the value of each radiomic feature in predicting the intracranial SD/PD of NSCLC patients receiving the second-line osimertinib therapy. **(C)** Correlation analysis of radiomic features before and after the removal of highly-correlated radiomics. NSCLC, non-small cell lung cancer; ICC, interclass correlation coefficients; SD, stable disease; PD, progressive disease.

We then investigated the predictive significance of MRI radiomic features in predicting the intracranial efficacy of the second-line osimertinib. After preprocessing of MRI images, 1,223 T1CE MRI radiomic features were extracted. These radiomic features were subsequently standardized by Combat harmonization and Z-score transformation. After ICC analysis, 1,191 (97.4%) features with ICC > 0.9 were retained for further analysis (Figure 2A). Univariate analysis indicated that 192 radiomic features were predictors of intracranial efficacy of the second-line osimertinib (Figure 2B), including 109 (56.8%) risk factors and 83 (43.2%) protective factors. We then applied the findCorrelation function in R package “caret” to filter out redundant radiomic features (Figure 2C). Initially, 25.65 and 16.67% of radiomic features shared a correlation coefficient (R) > 0.7 and R > 0.8, respectively, which dropped to 6.7 and 3.1% after we removed the high-correlated features. Eventually, 42 MRI radiomic features remained and subsequently subjected to the modeling analysis.

**Figure 3:**
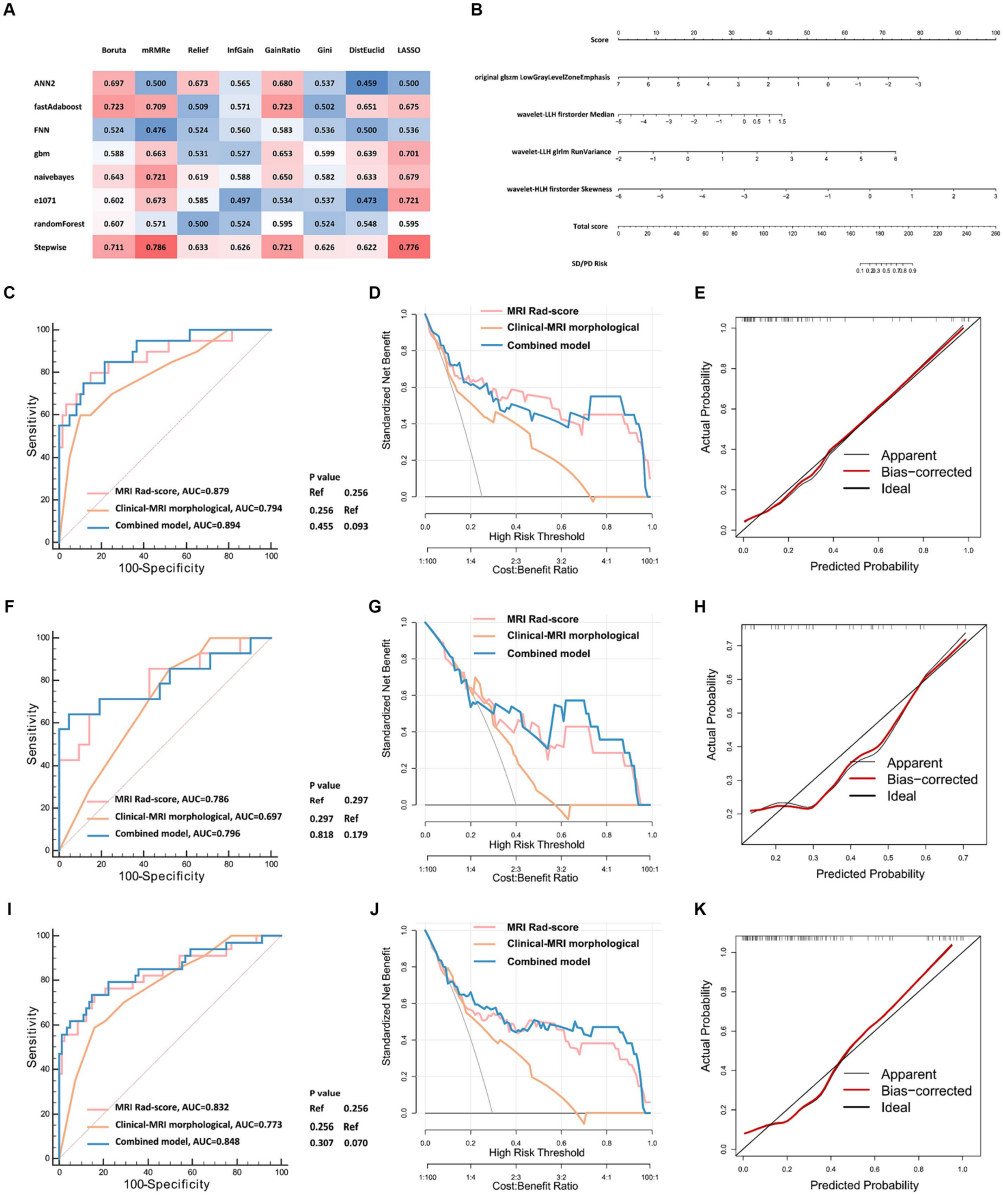
The development and validation of radiomic models predicting the intracranial efficacy of the second-line osimertinib. **(A)** Eight feature selectors and eight classifier machine learning algorithms were used to construct radiomic models. A nested 5-fold cross-validation strategy was applied. Eventually, 64 (8 × 8) combinations of radiomic feature selectors and classifiers were generated. The matrix displays the highest AUCs of ROC for the 5-fold validation. **(B)** Visualization of the optimal model constructed by the mRMR feature selector combined with the stepwise logistic regression classifier. **(C–K)** Model validation of the training **(C–E)**, validation **(F–H)**, and total cohort **(I–K)** using ROC curves, DCA, and calibration curves. ROC, receiver operator characteristic curve; DCA, decision curve analysis; AUC, area under the curve.

We then used the data of the training cohort to develop the machine learning model for predicting the intracranial efficacy of the second-line osimertinib. During this, we built a total of 64 machine learning models based on eight feature selectors and eight classifier algorithms. Ultimately, the model constructed using the mRMR feature selector and the stepwise logistic regression classifier achieved the highest average AUC in the 5-fold cross-validation, thereby establishing it as the final modeling approach (Supplementary Figure S2). The highest AUC from a single fold in the 5-fold cross-validation were 0.879 for the training cohort and 0.786 for the validation cohort. Consequently, this model was confirmed as the final model (Figure 3A). A nomogram was used to visualize the predictive model (Figure 3B). Each radiomic feature is assigned a score on its respective scale in the nomogram. To use it, find the patient’s value for each radiomic feature and draw a vertical line to the “Score” scale to get the individual score. Add up the scores for all features to obtain the “Total score.” Finally, use the “Total score” to draw a vertical line down to the “SD/PD Risk” scale to determine the probability of SD/PD.

Multivariate logistic regression analysis indicated that after the inclusion of the MRI radiomic model, clinical factors and MRI morphological features were no longer predictors of intracranial efficacy of the second-line osimertinib. In contrast, MRI radiomic model remained a robust independent predictor (Supplementary Table S2).

### Validation of the MRI radiomic model

We further validated the MRI radiomic model in different aspects (Figures 3C–K). The AUCs of the ROC curves of the MRI radiomic model were 0.879, 0.786, and 0.832 in the training, validation, and total cohort, respectively, which were remarkably higher than those of the clinical-MRI morphological model (Figures 3C,F,I). In the validation cohort, the model had 84.0% specificity and 73.5% sensitivity at the optimal threshold value. Besides, decision curve analysis showed that the MRI radiomic model had a higher net benefit than the clinical-MRI morphological model at various threshold probabilities, revealing the good clinical utility of the MRI radiomic model (Figures 3D,G,J). Furthermore, calibration curves also revealed satisfactory agreement between predictive and observational probabilities of poor intracranial efficacy (SD/PD) in the second-line osimertinib treatment (Figures 3E,H,K). However, incorporating clinical factors and MRI morphological features into the MRI radiomic model leaded to few improvements of the MRI radiomic model.

**Figure 4:**
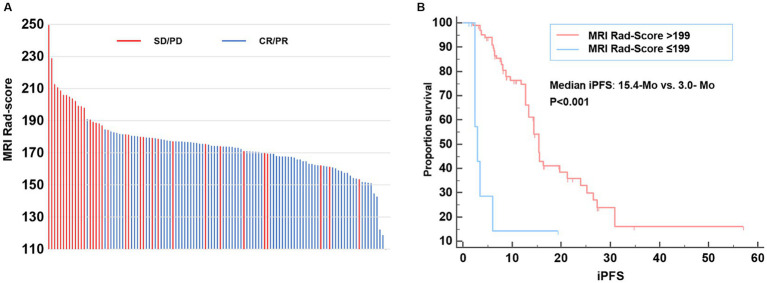
MRI Rad-score of each patient and model validation. **(A)** MRI Rad-score of each patient receiving second-line osimertinib therapy. **(B)** Kaplan–Meier curve exhibiting the iPFS of patients with MRI Rad-score > and ≤ 199. iPFS, intracranial progression-free survival.

As shown in Figure 4A, the MRI radiomic model could clearly distinguish NSCLC patients with favorable and poor intracranial efficacy in the second-line osimertinib treatment. Patients with a higher MRI radiomic score (Rad-score) are more prone to have a poor intracranial response (SD/PD). Brain MRI images of NSCLC patients with distinct Rad-score who achieved different intracranial response to osimertinib are shown in Supplementary Figure S3.

We further explored the relationship between MRI Rad-score and patients’ iPFS in the second-line osimertinib treatment. The X-tile analysis suggested that the optimal MRI Rad-score cutoff value for predicting iPFS was 199. The median iPFS of patients with MRI Rad-score > 199 and ≤ 199 were 3.0-Mo and 15.4-Mo, respectively (*p* < 0.001) (Figure 4B).

## Discussion

The brain is one of the most common metastatic organs of NSCLC. Approximately 25–30% of NSCLC patients develop brain metastases (3). Due to the limited blood–brain barrier permeability of first/s-generation EGFR-TKI agents, 34.2–52.9% of advanced NSCLC patients develop brain metastases during these treatments (37). As a third-generation EGFR-TKI agent, osimertinib targets and inhibits EGFR-sensitive and EGFR-T790M mutations with high selectivity. Notably, osimertinib has a high CNS activity (10) and crosses the blood–brain barrier more easily than the first/second-generation EGFR-TKIs (9, 10). In the current study, the intracranial ORR of second-line osimertinib treatment was 70.4%, which was similar to the results reported in the AURA, APOLLO, and OCEAN clinical trials (CNS-ORR: 54–71%) (14, 15, 17, 18, 38). The median iPFS in this study was 15.4 months, similar to the 11.7 months in the AURA3 trial (15). Collectively, it can be seen that in the real-world practice of the Chinese population, osimertinib still has a satisfactory effect on intracranial metastases of NSCLC patients. In this study, we firstly constructed and validated a brain MRI radiomic model that predicted the intracranial response of advanced NSCLC patients receiving second-line osimertinib.

Multiple brain metastases were present in 66.7% of cases in our study. Therefore, the objects of this study were brain metastases instead of individual patients. Currently, only very few studies investigated the ability of MRI radiomics to predict the intracranial efficacy of targeted therapy in lung cancer. Zhao et al. (24) performed a radiomic analysis of 87 brain metastases from 24 ALK-positive NSCLC patients treated with the ALK inhibitor ensatinib and established a model based on nine enhanced T1-weighted MRI radiological features that predicted the intracranial progression within 51 weeks (AUC = 0.85). The study of Chen et al. (25) found that the gradient boosting classifier models based on MRI radiomics and clinical factors had a tremendous predictive effect on the survival of brain metastatic NSCLC patients with EGFR, ALK, and/or KRAS mutation (AUC: 0.977, 0.905, and 0.947, respectively). Several researchers also reported that preoperative MRI radiomics of brain metastases from NSCLC patients could distinguish EGFR mutation status (29, 39). Besides, there were relatively many studies on MRI radiomics predicting the efficacy of gamma knife (21, 22) and stereotactic radiotherapy (23) for brain metastases of lung cancer. These studies, together with ours, demonstrated the ability of MRI radiomics to reflect the heterogeneity and biological characteristics of brain metastases and the feasibility of using MRI radiomics to predict the intracranial efficacy of various therapies such as targeted therapy, immunotherapy, and radiotherapy.

The MRI radiomic model constructed in this study consisted of four radiomic features, including two textural features (original glszm LowGrayLevelZoneEmphasis and wavelet-LLH glrlm RunVariance) and two histogram features (wavelet-LLH firstorder Median and wavelet-HLH firstorder Skewness). The histogram feature represented the overall distribution of grayscale and brightness information in the lesion area. On the other hand, GLCM and GLSZM were texture parameters that described the complexity, degree of variation, and texture thickness. Our study implied that differences in the biological behavior and response to osimertinib treatment of distinct brain metastases might be underlying the histogram and texture radiomic features. In a previous study, Wang et al. explored the use of MRI radiomics to predict the intracranial efficacy of first-and second-generation EGFR TKIs (27). Compared to our study, which utilized 1,223 radiomic features, their study used only 593 features. This difference is due to our use of a more comprehensive set of image preprocessing techniques and advanced feature extraction algorithms. This approach was designed to capture a wider range of image characteristics, providing a richer and potentially more informative dataset for model training and validation.

In terms of MRI morphological features, we found that peritumoral edema was a predictor of poor intracranial response to osimertinib treatment in patients with T790M-positive NSCLC, which is consistent with published literature (25, 40–42). Tini et al. (40) analyzed data of 42 NSCLC patients with 50 brain metastases and found that edema around the brain metastatic lesions was associated with unfavorable therapeutic response of radiosurgery. Patients with major edema had a poorer response to radiosurgery than cases with minor edema. Similar findings were reported by another research group (41). A recent study also showed that peritumoral edema predicted intracranial response to chemotherapy in NSCLC patients with multiple brain metastases (42). In addition, Chen et al. (25) reported that a lower edema/tumor ratio was related to a longer survival time in patients with brain metastatic NSCLC. The weakened response to chemoradiotherapy or targeted therapy in lesions with peritumoral edema may be related to the reduced blood–brain barrier permeability of drugs. Furthermore, interstitial fluid pressure from edema can hinder drug diffusion and weaken radiation beams. On the other hand, our study found that brain metastases with ring enhancement were less responsive to osimertinib than those without, which is also in line with previous reports (43). Lin et al. (43) showed that tumor necrosis, ring enhancement, and tumor anatomical location in brain MRI imaging are unfavorable prognosticators for cases treated with EGFR-TKI. They believed that neuroimaging features of tumor necrosis and ring enhancement implied rapid tumor growth, insufficient blood supply, and tissue hypoxia, which ultimately led to impaired drug penetration. Based on previously published reports and our findings, it can be seen that edema and ring enhancement around brain metastases have essential prognostic significance for NSCLC patients with brain metastasis.

This study had several limitations. Firstly, it is a retrospective study with a moderate sample size. Resultingly, shortcomings associated with its retrospective design could not be ruled out. Secondly, though internal validation was performed in this study, external validation using data from another medical center is lacking. Finally, since almost all patients in our center were of Asian race, the applicability of our novel radiomic model in other ethnic needs further exploration.

## Conclusion

The current study comprehensively explored the potential of MRI morphological features and MRI radiomics in predicting the intracranial efficacy of osimertinib treatment. The MRI radiomic model constructed in this study had a satisfactory predictive capability. This novel radiomic tool could help clinicians make personalized treatment strategies for brain metastatic NSCLC patients.

## Data Availability

The raw data supporting the conclusions of this article will be made available by the authors, without undue reservation.

## Footnotes

^1^ https://www.slicer.Org/

^2^ https://arxiv.org/abs/1612.07003

^3^ https://pyradiomics.readthedocs.io/en/latest/

^4^ https://github.com/Jfortin1/ComBatHarmonization
