# Supplementary material for: Enhancing intracranial efficacy prediction of osimertinib in non-small cell lung cancer: a novel approach through brain MRI radiomics
Source: Front Neurol. 2024 Aug 30;15:1399983. doi: 10.3389/fneur.2024.1399983 (PMC11395019; doi:10.3389/fneur.2024.1399983)
Supplement: Supplementary file 1 [file Table_1.DOCX]

Supplementary Material

**Supplementary Figures:**


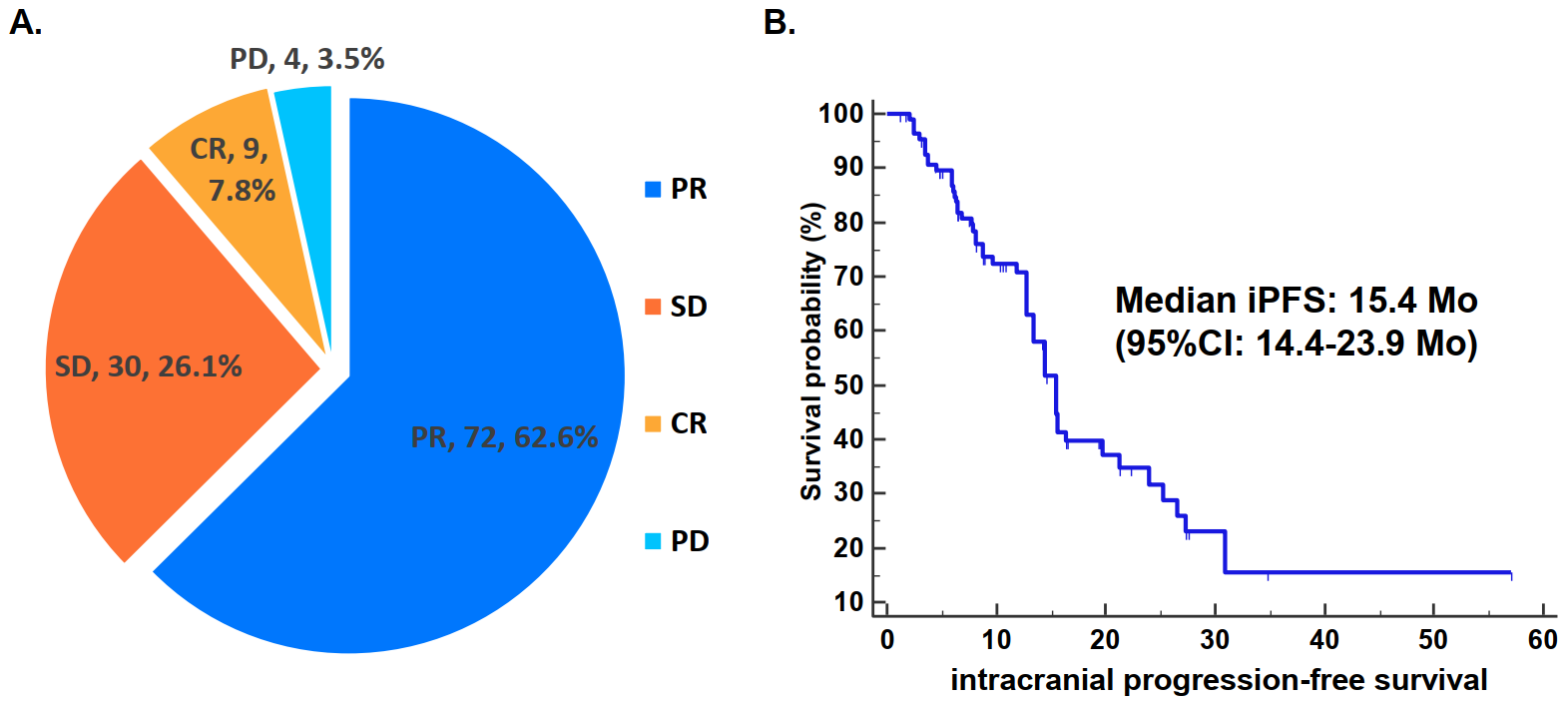


**Figure S1.** The overall intracranial efficacy of the second-line osimertinib. A. The proportion of NSCLC patients with intracranial response of CR, PR, SD, and PD. B. Kaplan-Meier curve exhibiting the iPFS of second-line osimertinib therapy.
NSCLC: non-small cell lung cancer, CR: complete remission, PR: partial remission, SD: stable disease, PD: progressive disease, iPFS: intracranial progression-free survival


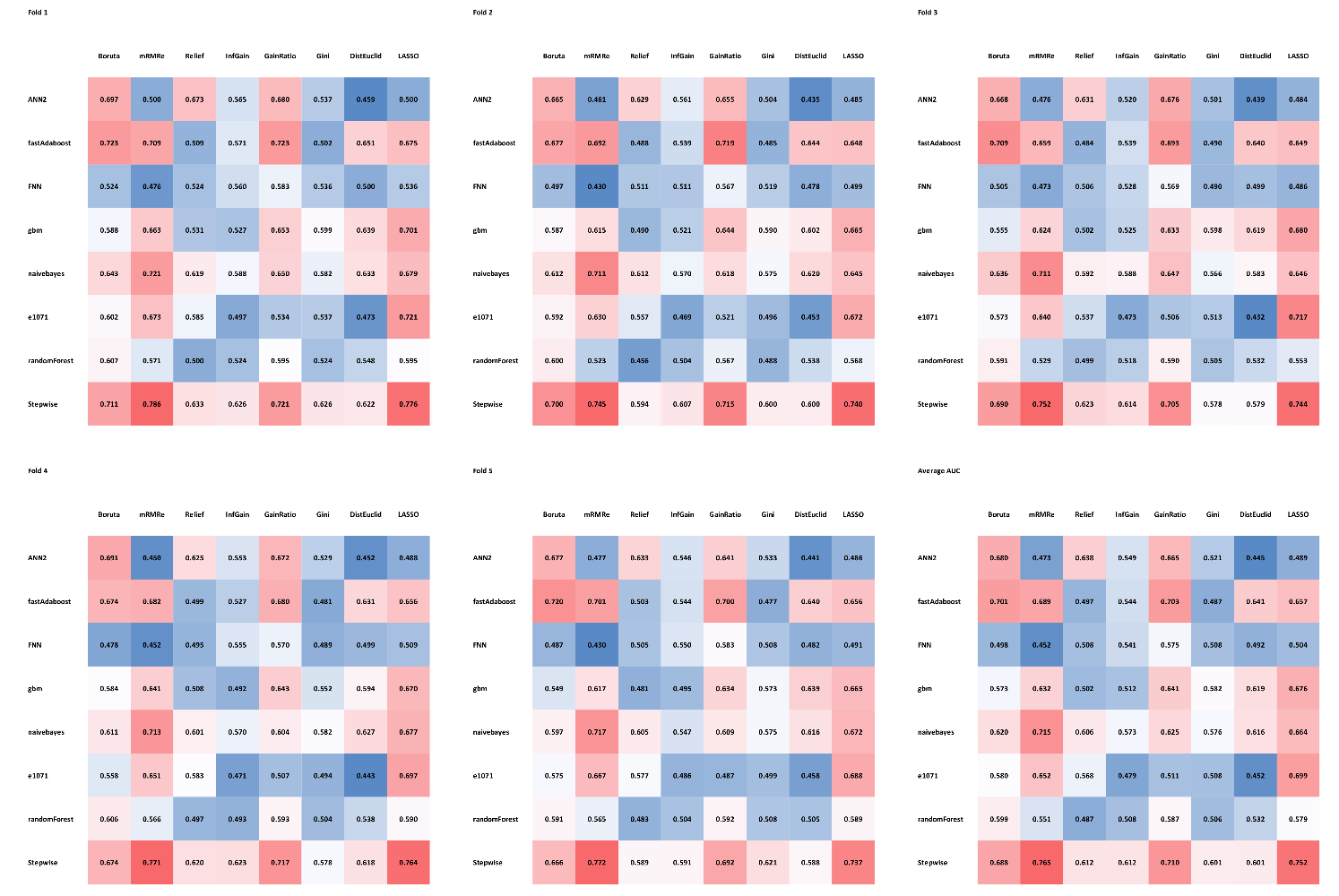


**Figure S2.** The 5-fold cross-validation results of the ROC's AUC are presented, with the final figure illustrating the average ROC AUC.

ROC: receiver operator characteristic curve; AUC: area under the curve


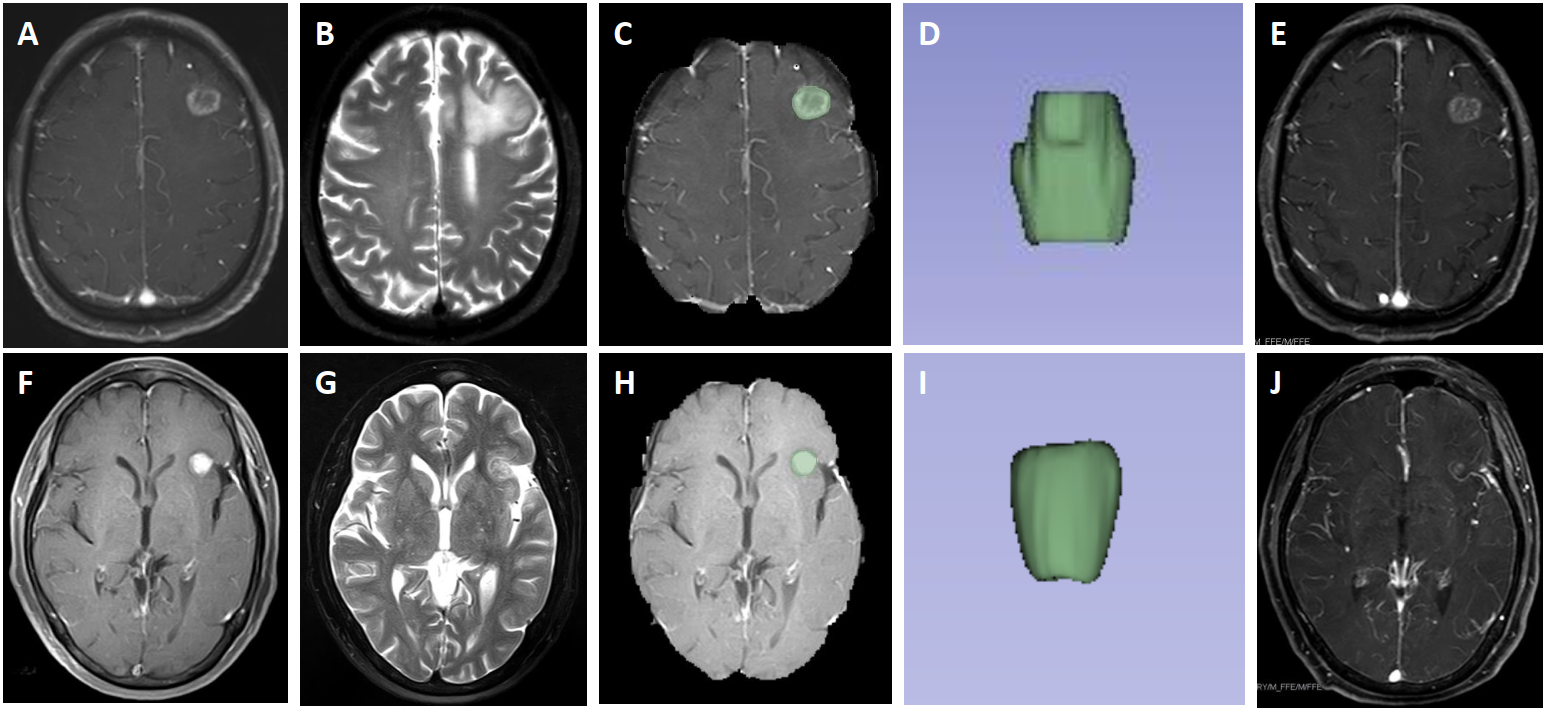


**Figure S3.** Brain MRI images of NSCLC patients who achieved different intracranial response to osimertinib. A-E: Patient 1, Rad-score: 249.7, intracranial response of osimertinib treatment: SD; F-J: Patient 2, Rad-score: 161.8, intracranial response of osimertinib treatment: PR

NSCLC: non-small cell lung cancer, SD: stable disease, PR: partial remission

**Supplementary Tables:**

**Table S1. Patients’ baseline characteristics corresponding to different BM in the training and validation cohort.**

|  | **Total BM** | |  | **BM of the Training cohort** | |  | **BM of the Validation cohort** | | ***P***  **Value** |
| --- | --- | --- | --- | --- | --- | --- | --- | --- | --- |
|  | **N** | **%** |  | **N** | **%** |  | **N** | **%** |  |
| **Age** |  |  |  |  |  |  |  |  |  |
| **>60** **Y** | 53 | 46.1% |  | 39 | 48.8% |  | 14 | 40.0% | 0.386 |
| **≤60 Y** | 62 | 53.9% |  | 41 | 51.3% |  | 21 | 60.0% |  |
| **Sex** |  |  |  |  |  |  |  |  |  |
| **Female** | 66 | 57.4% |  | 46 | 57.5% |  | 20 | 57.1% | 0.972 |
| **Male** | 49 | 42.6% |  | 34 | 42.5% |  | 15 | 42.9% |  |
| **Smoking history** |  |  |  |  |  |  |  |  |  |
| **No** | 100 | 87.0% |  | 69 | 86.3% |  | 31 | 88.6% | 0.734 |
| **Yes** | 15 | 13.0% |  | 11 | 13.8% |  | 4 | 11.4% |  |
| **ECOG-PS score** |  |  |  |  |  |  |  |  |  |
| **0** | 56 | 48.7% |  | 36 | 45.0% |  | 20 | 57.1% | 0.231 |
| **≥1** | 59 | 51.3% |  | 44 | 55.0% |  | 15 | 42.9% |  |
| **Initial EGFR mutation** |  |  |  |  |  |  |  |  |  |
| **L858R** | 53 | 46.1% |  | 38 | 47.5% |  | 15 | 42.9% | 0.832 |
| **19Del** | 51 | 44.3% |  | 34 | 42.5% |  | 17 | 48.6% |  |
| **Others** | 11 | 9.6% |  | 8 | 10.0% |  | 3 | 8.6% |  |
| **First-line EGFR-TKI drug** |  |  |  |  |  |  |  |  |  |
| **Gefitinib** | 62 | 53.9% |  | 42 | 52.5% |  | 20 | 57.1% | 0.372 |
| **Erlotinib** | 19 | 16.5% |  | 11 | 13.8% |  | 8 | 22.9% |  |
| **Icotinib** | 31 | 27.0% |  | 25 | 31.3% |  | 6 | 17.1% |  |
| **Afatinib** | 3 | 2.6% |  | 2 | 2.5% |  | 1 | 2.9% |  |
| **First-line EGFR-TKI PFS** |  |  |  |  |  |  |  |  |  |
| **<12 Mo** | 56 | 48.7% |  | 38 | 47.5% |  | 18 | 51.4% | 0.698 |
| **≥12 Mo** | 59 | 51.3% |  | 42 | 52.5% |  | 17 | 48.6% |  |
| **T stage** |  |  |  |  |  |  |  |  |  |
| **T1** | 19 | 16.5% |  | 11 | 13.8% |  | 8 | 22.9% | 0.233 |
| **T2** | 23 | 20.0% |  | 18 | 22.5% |  | 5 | 14.3% |  |
| **T3** | 9 | 7.8% |  | 3 | 3.8% |  | 6 | 17.1% |  |
| **T4** | 64 | 55.7% |  | 48 | 60.0% |  | 16 | 45.7% |  |
| **N stage** |  |  |  |  |  |  |  |  |  |
| **N0** | 24 | 20.9% |  | 21 | 26.3% |  | 3 | 8.6% | 0.575 |
| **N1** | 9 | 7.8% |  | 5 | 6.3% |  | 4 | 11.4% |  |
| **N2** | 44 | 38.3% |  | 26 | 32.5% |  | 18 | 51.4% |  |
| **N3** | 38 | 33.0% |  | 28 | 35.0% |  | 10 | 28.6% |  |
| **M stage** |  |  |  |  |  |  |  |  |  |
| **M1b** | 10 | 8.7% |  | 6 | 7.5% |  | 4 | 11.4% | 0.743 |
| **M1c** | 105 | 91.3% |  | 74 | 92.5% |  | 31 | 88.6% |  |
| **Stage** |  |  |  |  |  |  |  |  |  |
| **Ⅳa** | 10 | 8.7% |  | 6 | 7.5% |  | 4 | 11.4% | 0.743 |
| **Ⅳb** | 105 | 91.3% |  | 74 | 92.5% |  | 31 | 88.6% |  |
| **Maximum diameter of BM** |  |  |  |  |  |  |  |  |  |
| **>2cm** | 13 | 11.3% |  | 4 | 11.4% |  | 9 | 11.3% | 1.000 |
| **≤2cm** | 102 | 88.7% |  | 31 | 88.6% |  | 71 | 88.8% |  |
| **Ring enhancement** |  |  |  |  |  |  |  |  |  |
| **No** | 69 | 60.0% |  | 47 | 58.8% |  | 22 | 62.9% | 0.836 |
| **Yes** | 46 | 40.0% |  | 33 | 41.3% |  | 13 | 37.1% |  |
| **Peritumor edema** |  |  |  |  |  |  |  |  |  |
| **No** | 60 | 52.2% |  | 46 | 57.5% |  | 14 | 40.0% | 0.127 |
| **Yes** | 55 | 47.8% |  | 34 | 42.5% |  | 21 | 60.0% |  |

NSCLC: Non-Small Cell Lung Cancer; BM: brain metastases; PFS: Progression-free survival; EGFR: Epidermal Growth Factor Receptor; TKI: Tyrosine Kinase Inhibitor; ECOG-PS: Eastern Cooperative Oncology Group performance score

**Table S2. Features included in the MRI radiomic model and multivariate analysis for the second-line osimertinib therapy.**

| 1. **mRMR＆Stepwise logistic regression radiomic model** | | | |
| --- | --- | --- | --- |
|  | **OR** | **95% CI** | ***p* value** |
| **original glszm LowGrayLevelZoneEmphasis** | 0.38 | 0.15-0.95 | 0.038 |
| **wavelet-LLH firstorder Median** | 2.25 | 1.06-4.79 | 0.036 |
| **wavelet-LLH glrlm RunVariance** | 3.05 | 1.31-7.14 | 0.010 |
| **wavelet-HLH firstorder Skewness** | 3.86 | 1.55-9.64 | 0.004 |
| 1. **Multivariate analysis of MRI radiomic model, clinical and MRI morphological features** | | | |
|  | **OR** | **95% CI** | ***p* value** |
| **MRI radiomics model** | 1.11 | 1.05-1.17 | <0.001 |
| **Age：>60 vs. ≤60 Y** | 2.34 | 0.79-6.91 | 0.123 |
| **Ring enhancement: Yes vs. No** | 1.30 | 0.42-3.99 | 0.648 |
| **Peritumor edema: Yes vs. No** | 2.59 | 0.89-7.61 | 0.082 |

OR: Odds ratio; CI: Confidence interval
